
JOURNAL INFORMATION
==============================
NLM Title Abbreviation: ScientificWorldJournal
Journal ID: ScientificWorldJournal
NLM Journal ID: 101131163
Journal ID: TSWJ

The Scientific World Journal

ISSN: 2356-6140
EISSN: 1537-744X
Publisher: Wiley

ARTICLE INFORMATION
==============================
PMCID: PMC6009706
PMID: 29973830
DOI: 10.1100/tsw.2002.55
Article ID: 786269
Article version: 1
Subjects: Short Report

Synthetic Peptide Corresponding to The Amino-Terminal Region of the Human Tryptophanyl-Trna Synthetase, a Component Of Alzheimer’S Disease Special Congophlic Plaques Aggregates In Vitro to Form Amyloid-Like Fibrils

Paley Elena L. 1 *
Malinovskii Vladimir 2
Solomon Beka 3
Expert BioMed, Inc., 9257 Emerson Ave. Surfside, Miami, FL 33154, USA
Department of Biochemistry and Molecular Biology, University of Miami School of Medicine, PO Box 016129, Miami, FL 33101, USA
Department of Molecular Microbiology and Biotechnology, Tel Aviv University Faculty of Life Sciences, Tel Aviv 69978, Israel
*Elena L. Paley: elenapaley@cs.com
Publication date: 2002
Electronic publication date: 2002 May 3
Volume: 2
First page: 117
Last page: 118
Copyright: Copyright © 2002 Elena L. Paley et al.
Copyright year: 2002
License: This is an open access article distributed under the Creative Commons Attribution License, which permits unrestricted use, distribution, and reproduction in any medium, provided the original work is properly cited.
License URL: https://creativecommons.org/licenses/by/3.0/

==============================
Advances in Gene Technology: The Genome and Beyond –
Structural Biology for Medicine (Proceedings of the 2002 Miami
*Nature Biotechnology* Winter Symposium)
*TheScientificWorld* 2002, 2(S2), 117–118
ISSN 1532-2246; DOI 10.1100/tsw.2002.55

### SYNTHETIC PEPTIDE CORRESPONDING TO THE AMINO-TERMINAL REGION OF THE HUMAN TRYPTOPHANYL-tRNA SYNTHETASE, A COMPONENT OF ALZHEIMER'S DISEASE SPECIAL CONGOPHLIC PLAQUES AGGREGATES *IN VITRO* TO FORM AMYLOID-LIKE FIBRILS

Elena L. Paley*, Vladimir Malinovskii[¶], and Beka Solomon[#]
[*]Expert BioMed, Inc., 9257 Emerson Ave. Surfside, Miami, FL 33154; [¶]Department of Biochemistry and Molecular Biology, University of Miami School of Medicine, PO Box 016129, Miami, FL 33101; [#]Department of Molecular Microbiology and Biotechnology, Tel Aviv University Faculty of Life Sciences, Tel Aviv 69978, Israel
ElenaPaley@cs.com

**INTRODUCTION.** Tryptophanyl-tRNA synthetase (TrpRS, EC 6.1.1.2), a phosphoprotein[1] is a member of a family of aminoacyl-tRNA synthetases catalyzing the esterification of an amino acid to its cognate tRNA. TrpRS belongs to the class I synthetases sharing two amino acid consensus motifs HIGH and KMSKS. In addition, a conserved consensus domain of 46 amino acids called WHEP-TRS is present at the N-terminal extremity of TrpRS (amino acids 19 to 64 of the human TrpRS) in a number of higher eukaryotic aminoacyl-tRNA synthetases. This domain contains a central alpha-helical region (helix-turn-helix) and its role remains unclear. We have characterized TrpRS in human brain and revealed new type plaque-associated TrpRS in Alzheimer's disease brain sections. The peptides corresponding to TrpRS were synthesized and their aggregation behavior was analyzed.

**METHODS.** Monoclonal and polyclonal antibodies to human recombinant and bovine purified TrpRS were used for immunohistochemical analysis of brain sections of Alzheimer's diseases patients and controls. Two polypeptides corresponding to regions of N-terminal (19 amino acids) and C-terminal (24 amino acids) sequence of human TrpRS were synthesized. Congo Red staining and birefringence were examined for anti-TrpRS immunostained brain sections and synthetic peptides.

**RESULTS.** Plaque-like unusual formations were positively immunostained with monoclonal and polyclonal antibodies to TrpRS in brain sections of Alzheimer's disease patients (Fig. 1). The plaques bound Congo Red and showed birefringence (Fig. 1, low panel). No such plaques were detected in the sections not related to neurodegeneration.

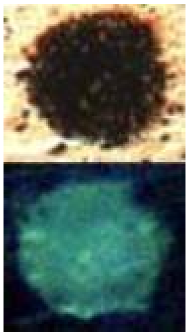

**FIGURE 1.** Congofilic plaque possessing TrpRS.

Fibrils in TrpRS-possessing plaques were revealed by confocal microscopy. Synthetic peptide corresponding to N-terminal region (32-50 residues) of hTrpRS spontaneously aggregated and formed fibrils. These fibrils bound Congo Red and showed birefringence, whereas C-terminal peptide (414-437 residues) did not bind Congo Red and remained in solution.

**DISCUSSION.** We suggest that antibodies to TrpRS stain new type congofilic plaque of Alzheimer's disease. The N-terminal TrpRS peptide (32-50) lies in WHEP-TRS consensus domain and shows a degree of similarity to peptide that exhibits fibrillogenic properties and related to neurodegenerative Creutzfeldt-Jacob disease[2]. These results lend support to a role for TrpRS in the development of neurodegenerative disease.

**REFERENCES**

1. Paley, E.L. (1997) Eur. J. Biochem. 244, 780–788.
2. Goldfarb, L.G., Brown, P., Haltia, M., Ghiso, J., Grangione, B., and Gajdusek, D.C. (1993) Proc. Natl. Acad. Sci. U. S. A. 90, 4451–4454.
